# Development and Validation of a Mutational Burden-Associated LncRNA Signature for Improving the Clinical Outcome of Hepatocellular Carcinoma

**DOI:** 10.3390/life11121312

**Published:** 2021-11-28

**Authors:** Mingjun Xu, Ting Ma, Shanping Shi, Jingjun Xing, Yang Xi

**Affiliations:** Diabetes Center, Zhejiang Provincial Key Laboratory of Pathophysiology, Institute of Biochemistry and Molecular Biology, School of Medicine, Ningbo University, Ningbo 315211, China; 1911074035@nbu.edu.cn (M.X.); 186001786@nbu.edu.cn (T.M.); 176001536@nbu.edu.cn (S.S.); xingjingjun@nbu.edu.cn (J.X.)

**Keywords:** mutational burden, long non-coding RNA, hepatocellular carcinoma, prognosis, TP53

## Abstract

Background: Long non-coding RNAs (lncRNAs) modulate numerous cellular processes, including DNA damage repair. Here, we investigated the clinical importance of lncRNAs associated with mutational burden in hepatocellular carcinoma (HCC). Methods: Prognosis-related lncRNAs associated with mutational burden were screened and determined to score the mutational burden-associated lncRNA signature (MbLncSig) from TCGA. Prognostic values and predictive performance of the MbLncSig score were analysed. Results: Four mutational burden-associated lncRNAs (AC010643.1, AC116351.1, LUCAT1 and MIR210HG) were identified for establishing the MbLncSig score. The MbLncSig score served as an independent risk factor for HCC prognosis in different subgroup patients. The predictive performance of one-year and three-year OS was 0.739 and 0.689 in the entire cohort, respectively. Moreover, the MbLncSig score can further stratify the patient survival in those with TP53 wild type or mutation. Conclusions: This study identified a four-lncRNA signature (the MbLncSig score) which could predict survival in HCC patient with/without TP53 mutation.

## 1. Introduction

Hepatocellular carcinoma (HCC), ranked sixth among the most common cancers, is the leading cause of tumour-related mortality worldwide [1]. Despite recent advancements in treatment strategies, such as surgical resection, liver transplantation, radiofrequency ablation, and target therapies, postoperative early recurrence and distant metastasis are considered as the major impediments to patient prognosis [2,3].

Somatic mutation is the basic condition for cancer development [4]. Tumor mutation burden (TMB) is defined as the number of somatic (such as missense, deletion, or insertion) mutations per megabase of genome examined [5]. In different types of cancer, there is a significant correlation between tumor mutational burden and immune-related adverse events [6]. It is of great significance to study the mutational burden for predicting patient survival. For hepatitis B virus (HBV)-associated HCC, the HBV DNA integrates into the host genome to induce genomic instability and direct insertional mutations in several cancer-related genes [7]. In some studies, whole-exome sequencing of hepatocellular carcinoma patients revealed a large number of genes with non-silencing mutations, which were related to cancer suppression or promotion, chromatin remodeling and anti-oxidation [8]. Protein–DNA interactions are widely believed to maintain genomic stability, but new research shows that RNA is also involved in regulating genome maintenance [9,10]. There is increasing evidence that many genomic mutations in cancer occur not in coding regions, but in the non-coding regions that transcribe long non-coding RAN [11]. Long non-coding RNA (lncRNA) molecules have length > 200 nucleotides. Increasing evidence has been suggesting the important role of lncRNAs in regulating genome maintenance [12,13,14]. A previous study reported that DNA damage results in the activation of a lncRNA [14]. Betts et al. reported that two lncRNAs (CUPID1 and CUPID2) participate in modulating pathway choice for the repair of double-strand breaks in hormone-receptor-positive breast tumours [12]. Bao et al. found that the gene signature of two lncRNAs related to genomic instability is associated with prognosis in breast cancer [15]. Detecting lncRNA has important therapeutic implications for genome integrity and treatment [16,17,18]. Although studies have explored a significant correlation between lncRNAs and genomic instability, mutational burden-associated lncRNAs in HCC remain unclear. Therefore, the identification of novel prognostic biomarkers related to mutational burden for HCC is significant for prognostic assessment and the development of therapeutic strategies.

In this study, we explored several mutational burden-associated lncRNAs based on the differential expression of lncRNAs between patients with high and low somatic mutation counts. Further, we established an lncRNA model to predict patient survival, which could contribute to the prognostic assessment.

## 2. Materials and Methods

### 2.1. Data and Patients

The mRNA sequencing data (FPKM) of 374 HCC tissues and 50 corresponding non-tumour tissues were extracted from The Cancer Genome Atlas (TCGA) (https://portal.gdc.cancer.gov/, accessed on 3 December 2020). In addition, clinicopathological characteristics, somatic mutation data, copy number variant data, and the survival information of the included patients were obtained from the database. The lncRNA expression data were extracted from the mRNA expression profile and name of the gene symbol was normalized using the GTF format. The included patients were allocated randomly into the training (n = 172) and testing (n = 171) cohorts. The training cohort was used to develop and establish the prognostic risk model, whereas the testing cohort was used to independently corroborate the model’s predictive performance.

### 2.2. Identification of Mutational Burden-Associated lncRNAs

To screen lncRNAs associated with mutational burden, we calculated the cumulative somatic mutation count of all patients and ranked the counts in order from the highest to lowest. The patients with a high cumulative somatic mutation count (top 25%) comprised the high mutational burden (HMb) group, whereas those with a low cumulative somatic mutation count (bottom 25%) comprised the low mutational burden (LMb) group. The Wilcoxon test was performed to screen the lncRNAs that were differentially expressed between the groups (*p* < 0.05 and |log2 (fold change)| > 1). The HCC samples in the TCGA cohort were submitted to unsupervised hierarchical clustering analysis based on the expression levels of the identified mutational burden-associated lncRNAs, and the patients were assigned either to the LMb group or to the HMb group.

The association between lncRNAs and interacted mRNAs was determined using Pearson correlation analysis, and the top 40 mRNAs were regarded as co-expression mRNAs. The Gene Ontology (GO) functional enrichment analysis of those co-expression mRNAs was conducted to predict the potential functions of lncRNAs.

### 2.3. Relationship between Mutational Burden and Genome Instability

Besides the somatic mutations, we also estimated the ratio of copy number variants (CNV) of each patient. The CNV data were retrieved from the TCGA database using the GDC download function from the TCGA biolinks R package. The copy number variation (CNV) pipeline uses Affymetrix SNP 6.0 array data to identify genomic regions that are repeated and infer the copy number of these repeats. Segments with focal CNV values smaller than −0.2 were categorized as “loss variants”. Segments with focal CNV values bigger than 0.2 were categorized as “gain variants”.

### 2.4. Establishment of a Mutational Burden-Related lncRNA Signature

To establish a mutational burden-related lncRNA predictive model, only patients with ≥30 days of survival or follow-up duration were selected. The correlation between mutational burden-associated lncRNA expression and overall survival (OS) was assessed using Cox proportional univariate and multivariate analyses. The sum of multivariate regression coefficients of mutational burden-associated lncRNAs and their expressions provided the mutational burden-associated lncRNA signature (MbLncSig) score. The median signature score of the training cohort was set as the risk cut-off value, and then, patients were assigned to either the high-risk or low-risk group based on the MbLncSig score.

### 2.5. Identification of the Clinicopathological Risk Parameters of HCC

Potential clinicopathological risk factors and the prognostic ability of the MbLncSig score were determined using the Cox regression model. Univariate and multivariate analyses included variables such as age, gender, histological grade, the American Joint Committee on Cancer (AJCC) stage, TNM stage, and MbLncSig score, and the analyses were performed in the training, testing and entire cohorts. Significant parameters identified in the univariate regression analysis were further included for validation in the multivariate analysis.

### 2.6. Statistical Analysis

Significant survival-related lncRNAs and clinical parameters were determined using univariate and multivariate Cox proportional hazard regression analyses. The Kaplan–Meier curve provided the prognostic value of the MbLncSig score. The time-dependent receiver operating characteristic (ROC) curve helped in validating the MbLncSig score’s predictive performance. All data were analysed using R (version 3.6.1, https://cloud.r-project.org/, accessed on 18 December 2020) and Strawberry Perl (version 5.32.0.1, https://strawberryperl.com/, accessed on 24 December 2020). A two-tailed *p* value of <0.05 indicated a significant difference.

## 3. Results

### 3.1. Identification of Mutational Burden-Related lncRNAs in HCC

**Figure 1:**
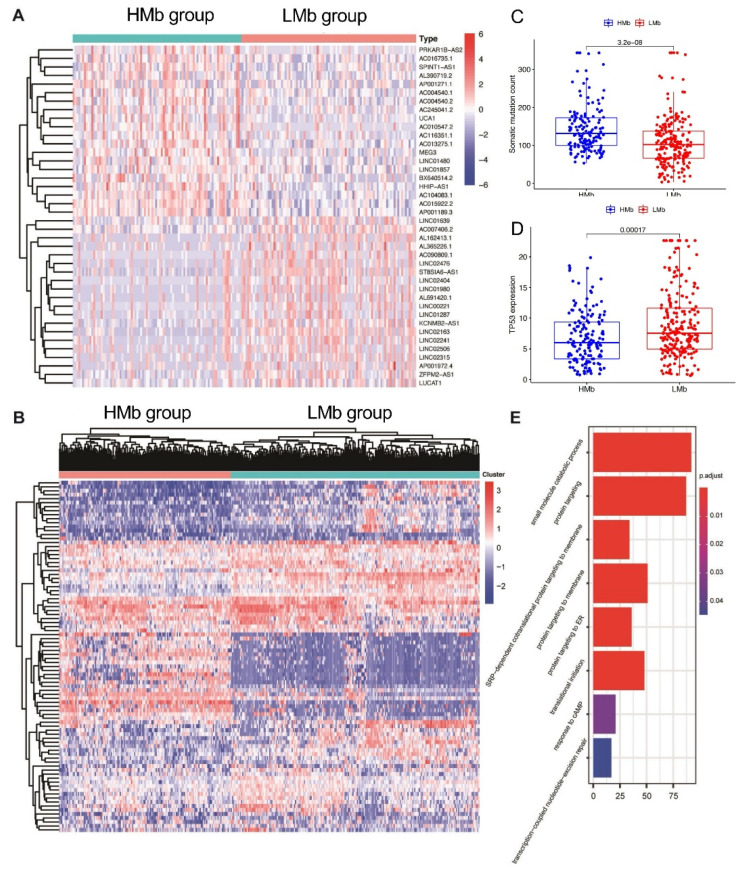
Identification of mutational burden-related lncRNAs in patients with hepatocellular carcinoma. (**A**) The heat map as an example of the expression of the top 20 identified mutational burden-associated lncRNAs. Differential expression analysis was performed between the patients with accumulative counts of high somatic mutation (the top 25%) (HMb group) and those with accumulative counts of low somatic mutation (the last 25%) (LMbgroup). (**B**) Unsupervised clustering analysis based on the identified 88 mutational burden-associated lncRNAs. (**C**) Boxplots of cumulative somatic mutation counts in the HMbgroup and the LMbgroup. (**D**) The expression of TP53 in the HMbgroup and the LMbgroup. (**E**) Biological process analyses for the identified lncRNAs correlated mRNAs.

The lncRNAs related to mutational burden were identified using the cumulative somatic mutation counts, which were calculated for each patient and ranked in order from the largest to the smallest. Patients with the top 25% (*n* = 93) and bottom 25% (*n* = 90) of the cumulative somatic mutation count were assigned to the HMb and LMb groups, respectively. The differential expression analysis of mutational burden-related lncRNAs between the groups revealed that 88 lncRNAs are differentially expressed, of which 56 are upregulated and 32 are downregulated. A detailed list of the differentially expressed lncRNAs is presented in Supplementary Table S1. A heatmap was drawn using the top 20 differentially expressed lncRNAs that were significantly upregulated or downregulated (Figure 1A). Subsequently, 374 HCC samples in the TCGA cohort, based on the expression levels of the 88 identified lncRNAs were analysed using the unsupervised hierarchical clustering approach (Figure 1B). Then, all the patients were allocated to either the HMb or LMb group.

First, the cumulative somatic mutation counts were compared between the HMb and LMb groups. As shown in Figure 1C, the HMb group displayed a significantly higher median value of the cumulative somatic mutation count than the LMb group (*p* < 0.001). Next, the TP53 expression level was compared between these groups. The tumour suppressor TP53 is a highly frequently mutated gene in HCC. Its expression was significantly upregulated in the LMb group compared with the HMb group (*p* < 0.001; Figure 1D).

In order to further investigate the correlation between mutational burden and genome instability, we included the copy number variant data from the TCGA cohort. We then estimated the gain ratio and loss ratio of each patient. We found that the patients with high mutational burden also tend to harbor more CNVs regardless of gain or loss with a significant statistical difference as shown in Figure S1. The copy number variants reflect the genomic instability. The significant correlation between mutational burden and CNV implies that the mutational burden can be a sort of indicator for genome instability.

Furthermore, we also investigated the mutation types and CNV ratios within the top 25 patients with higher mutational burden in Figure S2. As expected, the top 25% of patients with a high mutational burden present a higher frequency of mutations in most cancer driver genes, such as TP53 (35% vs. 9%). Besides, different mutation types are present in both groups. However, the splice_site, frame_shift_ins and in_framedel variants are only present in the top 25% of patients with a high mutational burden.

We then analysed the biological function of differentially expressed lncRNAs through detecting lncRNAs’ correlated protein-coding genes. The top 40 mRNAs were selected for GO analysis. The results showed that these mRNAs are correlated with gene mutation, such as response to cAMP and transcription-coupled nucleotide-excision repair, suggesting the potential functions of lncRNAs in gene mutation (Figure 1E).

### 3.2. Development and Validation of a Predictive Model for HCC Prognosis

To investigate the prognostic values of the 88 mutational burden-related lncRNAs, 343 patients with HCC (follow-up time or survival time: ≥30 days) from TCGA were allocated randomly to either the training (n = 172) or testing (n = 171) cohort. No statistical differences were observed in terms of age (*p* = 0.625), gender (*p* = 0.879), AJCC stage (*p* = 0.869), pathological grade (*p* = 0.518), T stage (*p* = 1.000), N stage (*p* = 0.966), and M stage (*p* = 0.932) between the training and testing cohorts (Supplementary Table S2).

The correlation between the 88 mutational burden-associated lncRNA expressions and the prognosis of HCC patients in the training cohort was assessed using the univariate Cox regression analysis. The finding revealed that eight mutational burden-associated lncRNAs are correlated significantly with prognosis (Table 1). Furthermore, the multivariate Cox regression analysis was performed to screen prognostic-related lncRNAs. Subsequently, AC010643.1, AC116351.1, LUCAT1, and MIR210HG were identified (Table 2). The predictive model was established using multivariable Cox analysis coefficients and expression levels of these four lncRNAs. Finally, the score was calculated using the following equation: MbLncSig = (0.360 × expression value of AC010643.1) + (0.209 × expression value of AC116351.1) + (0.227 × expression value of LUCAT1) + (0.156 × expression value of MIR210HG). The MbLncSig score was calculated for all patients in the training cohort. On the basis of the median MbLncSig score, these patients were classified into either the high-risk (≥ cut-off value) or low-risk (< cut-off value) group.

**Figure 2:**
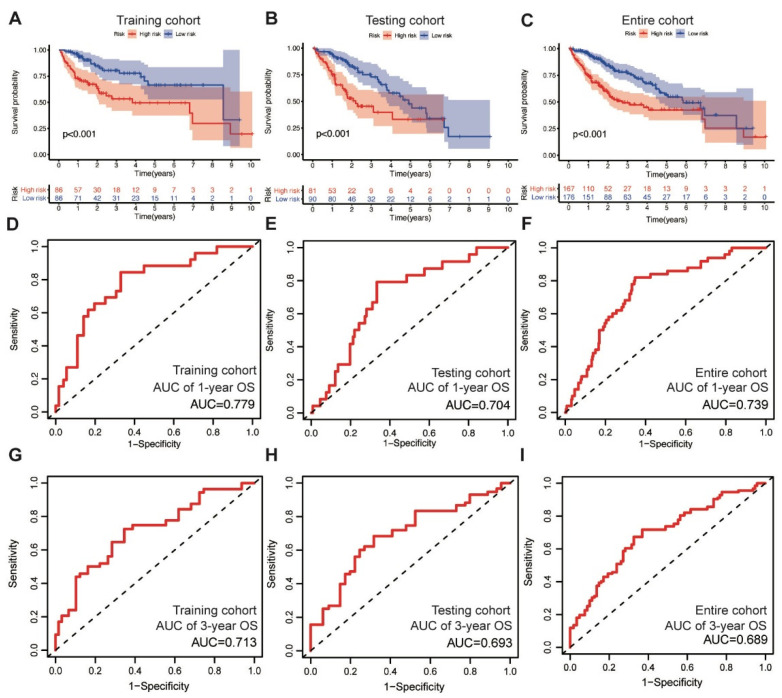
The prognostic values and predictive performance for patient survival of the mutational burden-associated lncRNA signature (MbLncSig) score. Kaplan-Meier curves of patient overall survival (OS) between the high-risk and low-risk groups in (**A**) the training cohort, (**B**) the testing cohort and (**C**) the entire cohort. Time-dependent ROC curve analyses in one-year OS by MbLncSig score (**D**) in the training cohort, (**E**) the testing cohort and (**F**) the entire cohort. Time-dependent ROC curves analysis in three-year OS by MbLncSig score (**G**) in the training cohort, (**H**) the testing cohort and (**I**) the entire cohort.

In the training cohort, the low-risk group displayed superior OS compared with the high-risk group, as revealed through the survival analysis (log-rank test, *p* < 0.001) (Figure 2A). We also validated the prognostic value of the MbLncSig score in the testing and entire cohorts, and a similar change pattern was observed between the groups (log-rank test, *p* < 0.001; Figure 2B,C). The time-dependent ROC curve analysis in the training, testing, and entire cohorts envisaged the predictive performance of the score for patient prognosis. The area under curves (AUCs) for one-year OS were 0.779, 0.704, and 0.739 in the training, testing, and entire cohorts, respectively (Figure 2D–F). For three-year OS, the respective AUCs were 0.713, 0.693, and 0.689 (Figure 2G–I). These results demonstrate the potential prognostic ability of the MbLncSig score for HCC.

### 3.3. MbLncSig Score as an Independent Risk Factor

Factors such as age, gender, AJCC stage, pathological grade, and the MbLncSig score were subjected to the univariate Cox regression analysis to identify the clinically prognostic variables for patients with HCC. Importantly, the MbLncSig score and AJCC stage were significantly associated with OS in the training cohort (Supplementary Table S3). The multivariate analysis further confirmed that the MbLncSig score and AJCC stage are the independent risk factors for the prognosis of HCC patients in the training cohort. Similarly, in the testing and entire cohorts, the multivariate analysis revealed the MbLncSig score and AJCC stage as independent risk factors for HCC prognosis.

**Figure 3:**
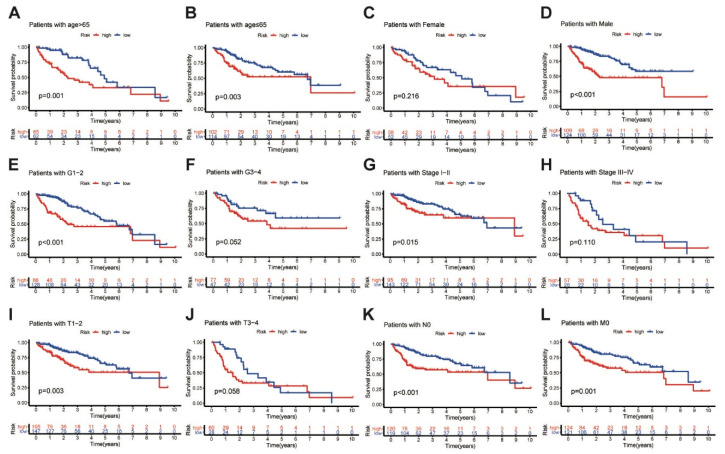
Kaplan-Meier curves analyses of overall survival in high-risk and low-risk groups among the patients with (**A**) age > 65 years old, (**B**) age ≤ 65 years old, (**C**) female, (**D**) male, (**E**) grade I-II, (**F**) grade III-IV (**G**) AJCC stage I-II, (**H**) AJCC stage III-IV, (**I**) T stage I-II, (**J**) T stage III-IV, (**K**) N stage 0 and (**L**) M stage 0, Tumor-Node-Metastasis, TNM.

In addition, further analyses were performed to determine the predictive ability of the MbLncSig score in different subgroups in all populations. Based on the MbLncSig score, the patients were classified into the high-risk or the low-risk group. Importantly, OS was significantly stratified between the risk groups in patients aged > 65 years (log-rank test, *p* = 0.001; Figure 3A), patients aged ≤ 65 (log-rank test, *p* = 0.003; Figure 3B), male patients (log-rank test, *p* < 0.001; Figure 3D), patients with pathological grade I-II (log-rank test, *p* < 0.001; Figure 3E), patients with AJCC stage I-II (log-rank test, *p* < 0.001; Figure 3G), patients with T stage I-II (log-rank test, *p* = 0.003; Figure 3I), patients with N stage 0 (log-rank test, *p* < 0.001; Figure 3K), and patients with M stage 0 (log-rank test, *p* = 0.001; Figure 3L). No differences were observed in female patients (log-rank test, *p* = 0.216; Figure 3C), patients with pathological grade III-IV (log-rank test, *p* = 0.052; Figure 3F), AJCC stage III-IV (log-rank test, *p* = 0.110; Figure 3H), or T stage III-IV (log-rank test, *p* = 0.058; Figure 3J). The findings revealed that the MbLncSig score serves as an independent prognostic component for patients with HCC.

### 3.4. Correlation with the Somatic Mutation in the Different Cohorts

**Figure 4:**
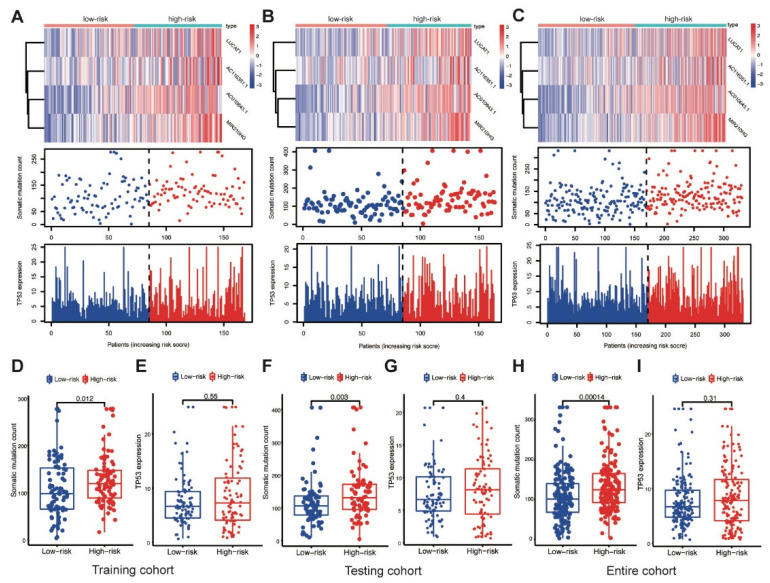
Performance assessment of MbLncSig score. The expression patterns of lncRNAs, distribution of somatic mutation counts and expression level of TP53 in the high-risk and low-risk groups. (**A**,**D**,**E**) in the training cohort, (**B**,**F**,**G**) in the testing cohort, (**C**,**H**,**I**) the entire cohort.

AC010643.1, AC116351.1, LUCAT1 and MIR210HG expression levels; somatic mutation counts; and TP53 expression levels were investigated in the training cohort. Elevated MbLncSig scores indicated the upregulation of those lncRNAs (Figure 4A). The somatic mutation counts were significantly higher in patients with a high score than in patients with a low score (*p* < 0.001), whereas no statistical difference was observed in TP53 expression levels between the high- and low-risk groups (Figure 4D,E). Expression patterns were also assessed in both the testing and entire cohorts. As shown in the Figure 4B,C, with an increase in the MbLncSig score, similar expression patterns were observed in both cohorts. Similarly, in both the cohorts, the high-risk group displayed a significantly higher number of somatic mutations than the low-risk group. However, no statistical differences were observed in TP53 expression levels between the groups in both cohorts (Figure 4F–I). These results indicated that the performance of MbLncSig score is superior in the training, testing, and entire cohorts.

### 3.5. Performance Comparison in Prognostic Prediction

**Figure 5:**
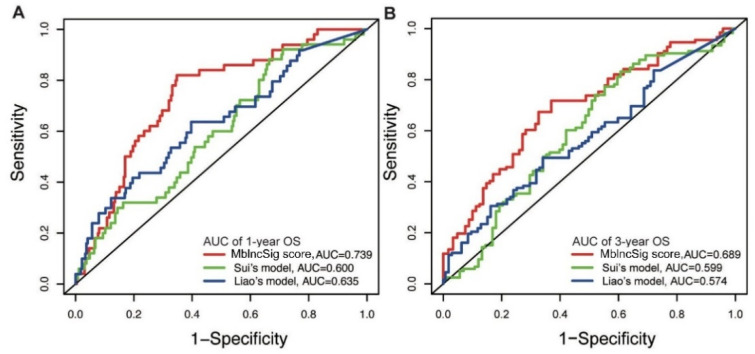
Comparison of predictive performance between our model and two published models. The time-dependent ROC curves of one-year (**A**) and three-year (**B**) overall survival among three models.

Next, the predictive performance for OS was compared between our model and two published lncRNA signatures. One predictive model, published by Sui’s study (Sui’s model), consisted of four lncRNA-related signatures using the TCGA cohort (LINC00261, TRELM3P, GBP1P1, and CDKN2B-AS1) [19]. Another predictive model, derived from Liao’s study (Liao’s model), included four lncRNA-related signatures (AC025016.1, LINC01164, LINC01183 and LINC01269) using the TCGA cohort [20]. Our results showed that the AUC of one year of our predictive model is 0.739, which is superior to those of Sui’s model (AUC: 0.600) and Liao’s model (AUC: 0.635) (Figure 5A). Similarly, the AUC of the three-year OS of our model is also higher than those of the two published models (AUC: 0.689 vs. 0.599 and 0.574) (Figure 5B). These findings indicated a strong performance of the MbLncSig score in predicting the prognosis of patients with HCC.

### 3.6. Prognostic Stratification Based on MbLncSig Score and TP53 Status

**Figure 6:**
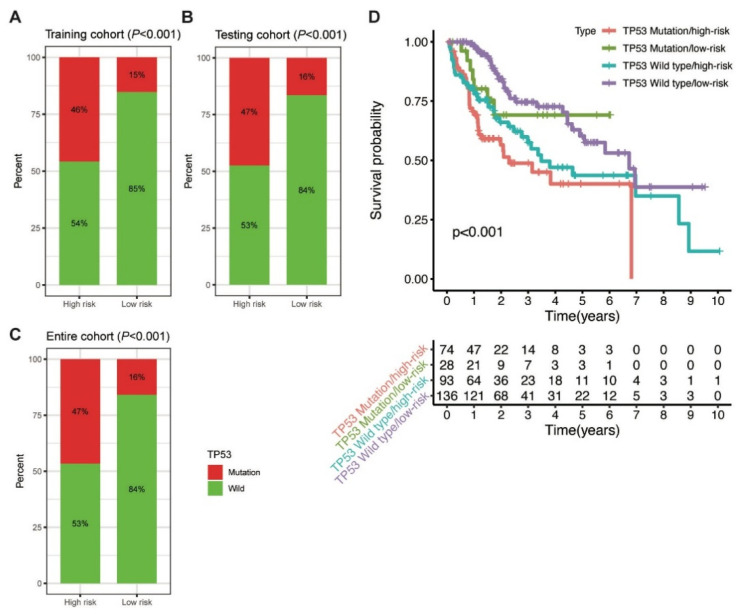
Correlation between the MbLncSig score and TP53 mutational status. (**A**) The proportion of TP53 mutation between the high-risk and low-risk groups in the training cohort. (**B**) The proportion of TP53 mutation between the high-risk and low-risk groups in the testing cohort. (**C**) The proportion of TP53 mutation between the high-risk and low-risk groups in the entire cohort. (**D**) Kaplan-Meier curve analyses of overall survival for patients with different MbLncSig scores and TP53 mutational status.

Further, the proportion of TP53 mutations was analysed between the high-risk and low-risk groups. In the training cohort, TP53 mutation was detected in 46% and 15% of patients in the high-risk and low-risk groups, respectively (*p* < 0.001; Figure 6A). Similarly, a higher percentage of TP53 mutation was observed in the high-risk group than in the low-risk group in the testing (47% vs. 16%, *p* < 0.001) and entire (47% vs. 16%, *p* < 0.001; Figure 6B,C) cohorts. The findings indicated that the MbLncSig score is significantly correlated with TP53 mutation.

Considering the role of TP53 in maintaining genomic stability and its prognostic impaction, we further examined the prognostic value of MbLncSig score in the TP53 wild type and mutation populations. Intriguingly, survival analyses revealed that a significant difference was observed in the patients with TP53 wild type/low-risk, patients with TP53 wild type/high-risk, patients with TP53 mutation/low-risk and those with TP53 mutation/high-risk, suggesting the potential value of MbLncSig score among the patients with TP53 wild-type or mutation (Figure 6D). These findings suggested a promisingly prognostic value of the MbLncSig score for HCC patients and it can further stratify OS in HCC patients with TP53 wild-type and mutation.

## 4. Discussion

Recently, a tremendous amount of work has been carried out in the prognostic prediction of patients with HCC. Traditional staging systems, such as the Barcelona Clinic Liver Cancer (BCLC), the AJCC, the Tumor-Node-Metastasis (TNM) and the China Liver Cancer (CNLC) staging systems, have been developed for prognostic assessment based on the clinical and pathological characteristics [21,22,23]. However, the oncological outcomes of HCC patients remain heterogeneous considering the limited values of clinical characteristics [2]. Mutational burden is a common hallmark of most cancer. HBV infection, one of the leading risk factors in the carcinogenesis of HCC in China, could induce genetic mutations [7]. However, it is challenging to measure the degree of mutational burden. Many studies have revealed that the abnormal changes in transcriptome or epigenome contribute to the gene mutation [24,25]. A series of recent research has reported significant values of lncRNAs in genome maintenance [12,13,14]. Although some efforts have been made, the molecular mechanisms and clinical values of lncRNAs relevant with mutational burden in HCC are still unclear.

In the present study, we singled out 88 mutational burden-associated lncRNAs by comparing the lncRNA expression in patients with high somatic mutation counts and patients with low somatic mutation counts. GO function enrichment analysis revealed that lncRNAs associated mRNAs play an important role in response to cAMP and transcription-coupled nucleotide-excision repair. cAMP signaling pathway was associated with DNA replication stress and mutational burden [26]. The transcription-coupled nucleotide-excision repair could promote DNA damage accumulation and gene mutation [27]. Protein targeting was one of the significant functions in GO analysis, which indicated the potential indirect effects of mRNAs in the mutational burden, while still needing further studies.

A prognostic MbLncSig score was subsequently developed to predict HCC patient survival based on four hub lncRNAs (AC010643.1, AC116351.1, LUCAT1 and MIR210HG). Patients with high risk were associated with a dismal prognosis in the training cohort, which was identified in the testing cohort. The time-dependent one-year and three-year AUC showed satisfactory predictive performance for HCC patient prognosis in the training, testing and entire cohorts. These findings suggest the potential values of our predictive model for prognostic assessment in HCC patients.

Among the four hub lncRNAs, LUCAT1 appears to be a potential factor for HCC diagnosis and treatment [28]. It can regulate the ubiquitination and stability of DNA methyltransferase 1 (DNMT1), contributing to tumorigenesis in oesophageal squamous cell carcinoma [29]. DNA methyltransferases are associated with DNA repair or modification mechanisms [30]. A recent study reported that the alternative splicing of certain DNA damage-related genes is altered in colorectal cancer cells, following LUCAT1-facilitated interaction of those genes with PTBP1 [31]. In addition, a previous study showed that MIR210HG can promote tumor progression in cervical cancer, invasive breast cancer, HCC, colorectal adenocarcinoma and osteosarcoma [32,33,34,35,36]. Kang et al. reported that MIR210HG can promote proliferation and invasion through upregulating the methylation of CACNA2D2 promoter via binding to DNMT1 in non-small cell lung cancer [37]. These findings suggest potential values of LUCAT1 and MIR210HG in genome maintenance, regulating tumor progression and influencing HCC patient survival.

Furthermore, the correlation between the MbLncSig score and cumulative somatic mutation counts was determined. The results revealed that the MbLncSig scores are related to the cumulative somatic mutation counts in the training, testing, and entire cohorts. As a commonly altered gene in cancer, TP53 mutation was correlated significantly with poor survival in patients with HCC [38,39]. Therefore, prognostic assessment of HCC patients with TP53 mutation is clinically important to explore additional treatment options [39]. Our results demonstrated that the frequency of TP53 mutation is increased in the high-risk group compared with the low-risk group, suggesting a synergistic effect of the MbLncSig score and TP53 mutational status. Moreover, we determined the predictive potential of the MbLncSig score between patients with wild-type and mutated TP53. Interestingly, the MbLncSig score revealed different survival rates between patients with wild-type and mutated TP53, indicating the predictive ability of the score to identify patient prognosis based on the gene’s mutational status. Therefore, integrating the MbLncSig score with TP53 mutational status can provide new insights into the personalised risk stratification of patients with HCC.

The greatest limitation of the study is that the predictive model was established based on a single TCGA database. Further validation by other independent databases should be performed. However, because of the limited availability of the lncRNAs of HCC samples in GEO dataset, we did not use the GEO dataset for further validation. Functional studies are required to further investigate the mechanism of the MbLncSig both in vivo and in vitro.

In conclusion, we identified the MbLncSig score as an independent risk factor for stratifying the survival of patients in different subgroups. In addition, MbLncSig score could further distinguish between the prognosis in patients with or without TP53 mutation, which may contribute to prognosis assessment and further clinical decision-making in HCC patients.

## Figures and Tables

**Table 1:** Univariate Cox regression analysis of eight mutational burden-associated lncRNAs for overall survival in HCC patients.

Gene Symbol	HR (95% CI)	*p* Values
AC010643.1	1.501 (1.046–2.154)	0.027
AC116351.1	1.212 (1.058–1.388)	0.005
LUCAT1	1.354 (1.125–1.629)	<0.001
ZFPM2–AS1	1.080 (1.026–1.137)	0.003
AC245041.2	1.075 (1.017–1.137)	0.011
PRRT3–AS1	1.094 (1.025–1.167)	0.007
AC145343.1	1.422 (1.027–1.969)	0.034
MIR210HG	1.213 (1.107–1.330)	<0.001

**Table 2:** Multivariate Cox regression analysis was performed to screen prognostic-related lncRNAs.

LncRNA	Coef	*p* Values
AC010643.1	0.360299397	0.06199
AC116351.1	0.209283496	0.00529
LUCAT1	0.227065913	0.02114
MIR210HG	0.156374734	0.00386

## Data Availability

Not applicable.

## Supplementary Materials

The following are available online at https://www.mdpi.com/article/10.3390/life11121312/s1, Table S1: Differentially expressed lncRNAs; Table S2: Baseline characteristics of patients in the training, testing and entire cohorts; Table S3: Univariate and multivariate Cox regression analysis based on different clinical characteristics and overall survival in HCC patients. Figure S1: Copy number variants correlated with the mutational burden; Figure S2: Mutation frequency and type within two groups.
