# Supplementary material for: Development and Validation of a Mutational Burden-Associated LncRNA Signature for Improving the Clinical Outcome of Hepatocellular Carcinoma
Source: Life (Basel). 2021 Nov 28;11(12):1312. doi: 10.3390/life11121312 (PMC8706720; doi:10.3390/life11121312)
Supplement: Supplementary file 1 [file life-11-01312-s001.zip › life-1490875-supplementary.pdf]

life

MDPI

Article

Development and Validation of a Mutational Burden-Associated LncRNA Signature for Improving the Clinical Outcome of Hepatocellular Carcinoma

Mingjun Xu, Ting Ma, Shanping Shi, Jingjun Xing and Yang Xi *

Diabetes Center, Zhejiang Provincial Key Laboratory of Pathophysiology, Institute of Biochemistry and Molecular Biology, School of Medicine, Ningbo University, Ningbo 315211, China; 1911074035@nbu.edu.cn (M.X.); 186001786@nbu.edu.cn (T.M.); 176001536@nbu.edu.cn (S.S.); xingjingjun@nbu.edu.cn (J.X.)

* Correspondence: xiyang@nbu.edu.cn; Tel.: +86-574-87600754

Supplementary materials

Table S1. Differential expressed lncRNAs.

LncRNA name	Log10 (Fold Chang)	*P values*	Regulation
AP001972.4	5.702	0.001	Up
AL365226.1	3.343	0.006	Up
AL162413.1	2.617	< 0.001	Up
LINC01980	2.513	< 0.001	Up
AC090809.1	2.425	< 0.001	Up
LINC01287	2.401	0.003	Up
LINC02163	2.326	< 0.001	Up
AL691420.1	2.192	< 0.001	Up
LUCAT1	2.047	0.003	Up
KCNMB2-AS1	2.045	< 0.001	Up
ST8SIA6-AS1	2.010	< 0.001	Up
LINC02476	1.891	< 0.001	Up
ZFPM2-AS1	1.890	< 0.001	Up
AC007406.2	1.874	< 0.001	Up
LINC02241	1.792	< 0.001	Up
LINC00221	1.709	0.001	Up
LINC02315	1.691	< 0.001	Up
LINC01639	1.689	0.008	Up
LINC02506	1.547	0.001	Up
LINC02404	1.537	< 0.001	Up
AP000593.3	1.497	0.014	Up
LINC01139	1.494	< 0.001	Up
LINC01419	1.457	< 0.001	Up
CASC9	1.422	< 0.001	Up
BX649601.1	1.369	0.002	Up
AC096996.2	1.355	< 0.001	Up
LINC01124	1.346	< 0.001	Up
AC090015.1	1.292	< 0.001	Up
AC004862.1	1.264	0.003	Up
AL109917.1	1.244	< 0.001	Up
AL023803.1	1.230	< 0.001	Up
AC010643.1	1.226	< 0.001	Up

AL512353.1	1.219	< 0.001	Up
AC004080.2	1.193	0.002	Up
AC145343.1	1.170	< 0.001	Up
AC092490.1	1.159	< 0.001	Up
AL731684.1	1.156	0.004	Up
AP003119.1	1.148	0.001	Up
AC002456.1	1.146	< 0.001	Up
LINC01608	1.141	< 0.001	Up
Z73429.1	1.120	0.006	Up
DCXR-DT	1.109	0.001	Up
Z82246.1	1.105	0.005	Up
AC254562.2	1.105	0.005	Up
AP003119.2	1.093	< 0.001	Up
AC008035.1	1.089	< 0.001	Up
AC026462.3	1.088	0.001	Up
MIR210HG	1.079	0.002	Up
LINC01151	1.067	< 0.001	Up
AC016405.3	1.053	< 0.001	Up
PRRT3-AS1	1.053	< 0.001	Up
AP003174.1	1.044	0.008	Up
AC010205.1	1.032	0.003	Up
LINC01474	1.026	0.001	Up
AL512408.1	1.017	< 0.001	Up
AC079949.2	1.008	0.001	Up
AL133355.1	−1.045	< 0.001	Down
IGF2-AS	−1.074	0.001	Down
AL035661.1	−1.100	< 0.001	Down
AP000424.1	−1.121	0.008	Down
LINC02298	−1.181	< 0.001	Down
AGAP2-AS1	−1.190	< 0.001	Down
PRR26	−1.213	< 0.001	Down
AC015922.3	−1.232	< 0.001	Down
AP000757.2	−1.250	< 0.001	Down
AL355102.4	−1.297	< 0.001	Down
LINC00924	−1.331	< 0.001	Down
AP000424.2	−1.373	< 0.001	Down
MEG3	−1.408	< 0.001	Down
AL390719.2	−1.441	< 0.001	Down
AC104083.1	−1.444	< 0.001	Down
LINC01857	−1.453	0.001	Down
HHIP-AS1	−1.601	< 0.001	Down
AP001189.3	−1.779	< 0.001	Down
AP001271.1	−1.791	0.006	Down
AC004540.2	−1.888	< 0.001	Down
AC013275.1	−1.922	< 0.001	Down
AC015922.2	−1.979	< 0.001	Down
SPINT1-AS1	−2.047	< 0.001	Down
AC116351.1	−2.261	< 0.001	Down
LINC01480	−2.266	0.013	Down
BX640514.2	−2.354	0.001	Down
AC016735.1	−2.426	< 0.001	Down

AC004540.1	−2.447	0.002	Down
PRKAR1B-AS2	−2.658	0.001	Down
AC245041.2	−3.015	0.007	Down
AC010547.2	−3.422	< 0.001	Down
UCA1	−5.203	< 0.001	Down

Table S2. Baseline characteristics of patients in the training, testing and entire cohorts.

Covariates	Subgroup	Entire cohort, n (%)	Training cohort, n (%)	Testing cohort, n (%)	*P value*
Age	≤ 65	216 (62.97%)	111 (64.53%)	105 (61.4%)	0.625
	> 65	127 (37.03%)	61 (35.47%)	66 (38.6%)	
Gender	Female	110 (32.07%)	54 (31.4%)	56 (32.75%)	0.879
	Male	233 (67.93%)	118 (68.6%)	115 (67.25%)	
Pathological grade	Grade 1–2	214 (62.39%)	111 (64.53%)	103 (60.23%)	0.518
	Grade 3–4	124 (36.15%)	59 (34.3%)	65 (38.01%)	
	Unknow	5 (1.46%)	2 (1.16%)	3 (1.75%)	
AJCC stage	Stage I–II	238 (69.39%)	122 (70.93%)	116 (67.84%)	0.869
	Stage III–IV	83 (24.2%)	41 (23.84%)	42 (24.56%)	
	Unknow	22 (6.41%)	9 (5.23%)	13 (7.6%)	
T stage	T stage 1–2	252 (73.47%)	126 (73.26%)	126 (73.68%)	1.000
	T stage 3–4	88 (25.66%)	44 (25.58%)	44 (25.73%)	
	Unknow	3 (0.87%)	2 (1.16%)	1 (0.58%)	
N stage	N stage 0	239 (69.68%)	123 (71.51%)	116 (67.84%)	0.966
	N stage 1	3 (0.87%)	1 (0.58%)	2 (1.17%)	
	Unknow	101 (29.45%)	48 (27.91%)	53 (30.99%)	
M stage	M stage 0	245 (71.43%)	129 (75%)	116 (67.84%)	0.932
	M stage 1	3 (0.87%)	1 (0.58%)	2 (1.17%)	
	Unknow	95 (27.7%)	42 (24.42%)	53 (30.99%)	

The American Joint Committee on Cancer, AJCC; Tumor-node-metastasis, TNM.

Table S3. The different cohorts were analyzed using univariate and multivariate models.

Variables		Univariable model		Multivariable model	
		HR (95% CI)	*P-value*	HR (95% CI)	*P-value*
**Training cohort**					
Age		0.993 (0.972–1.015)	0.553		
Gender	Male and Female	0.718 (0.398–1.293)	0.270		
Grade	I, II, III and IV	1.165 (0.793–1.712)	0.435		
Stage	I, II, III and IV	1.903 (1.370–2.643)	< 0.001	1.703 (1.209–2.400)	0.002
GILncSig Score		1.397 (1.227–1.591)	< 0.001	1.321 (1.147–1.521)	< 0.001
**Testing cohort**					
Age		1.014 (0.994-1.036)	0.173		
Gender	Male and Female	0.812 (0.479-1.376)	0.439		
Grade	I, II, III and IV	1.065 (0.747-1.517)	0.728		
Stage	I, II, III and IV	1.777 (1.334-2.365)	< 0.001	1.753 (1.311–2.345)	< 0.001
GILncSig Score		1.091 (1.030-1.156)	0.003	1.084 (1.020–1.151)	0.009
**Entire cohort**					
Age		1.005 (0.991–1.020)	0.481		
Gender	Male and Female	0.758 (0.513–1.118)	0.162		
Grade	I, II, III and IV	1.121 (0.865–1.454)	0.388		
Stage	I, II, III and IV	1.808 (1.463–2.234)	< 0.001	1.763 (1.422–2.186)	< 0.001
GILncSig Score		1.116 (1.067–1.168)	< 0.001	1.104 (1.052–1.159)	< 0.001

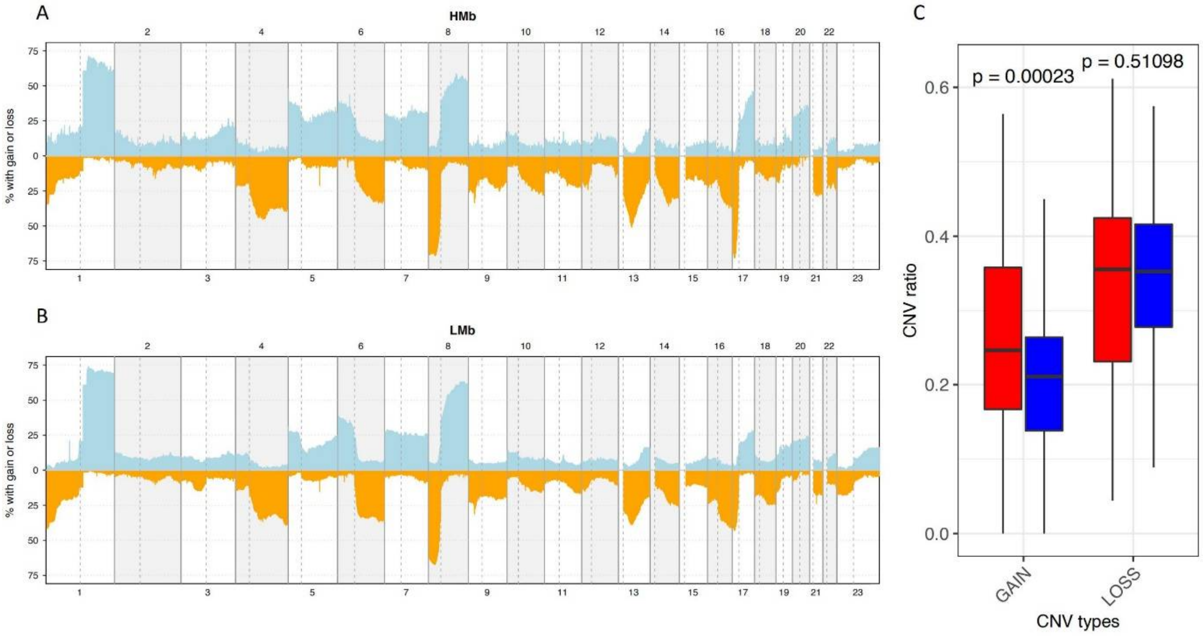

Figure S1. Copy number variants correlated with the mutational burden (A) The CNV ratio of patients with the high mutational burden (HMb). (B) The CNV ratio of patients with a low mutational burden (LMb). (C) The Comparison of CNV ratio between HMb and LMb patient

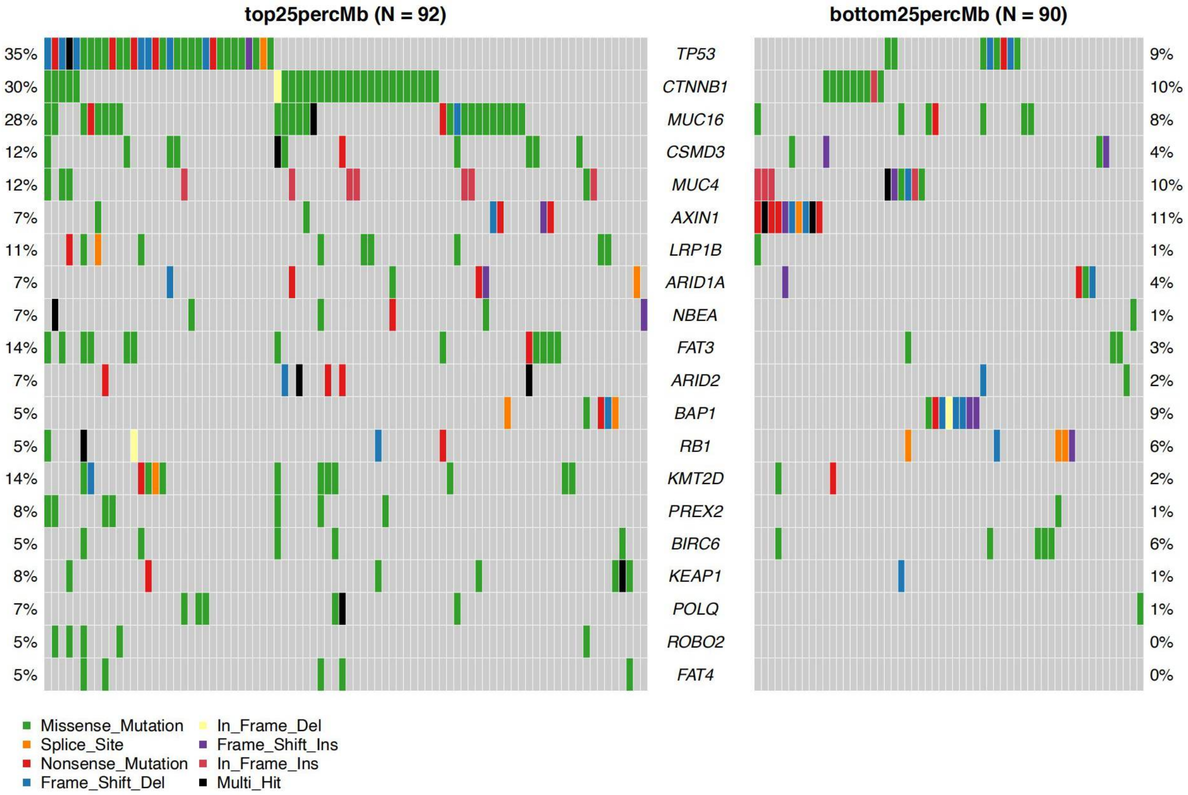

Figure S2. Mutation frequency and type within two groups
